# Impact of Spasticity on Balance Control during Quiet Standing in Persons after Stroke

**DOI:** 10.1155/2017/6153714

**Published:** 2017-09-14

**Authors:** Reza Rahimzadeh Khiabani, George Mochizuki, Farooq Ismail, Chris Boulias, Chetan P. Phadke, William H. Gage

**Affiliations:** ^1^Spasticity Research Program, West Park Healthcare Centre, Toronto, ON, Canada; ^2^School of Kinesiology and Health Science, York University, Toronto, ON, Canada; ^3^Heart and Stroke Foundation Canadian Partnership for Stroke Recovery, Sunnybrook Research Institute, Toronto, ON, Canada; ^4^Hurvitz Brain Sciences Research Program, Sunnybrook Research Institute, Toronto, ON, Canada; ^5^Department of Physical Therapy, University of Toronto, Toronto, ON, Canada; ^6^Division of Physiatry, University of Toronto, Toronto, ON, Canada; ^7^Faculty of Health, York University, Toronto, ON, Canada; ^8^Toronto Rehabilitation Institute, Toronto, ON, Canada

## Abstract

**Background:**

Balance impairments, falls, and spasticity are common after stroke, but the effect of spasticity on balance control after stroke is not well understood.

**Methods:**

In this cross-sectional study, twenty-seven participants with stroke were divided into two groups, based on ankle plantar flexor spasticity level. Fifteen individuals with high spasticity (Modified Ashworth Scale (MAS) score of ≥2) and 12 individuals with low spasticity (MAS score <2) completed quiet standing trials with eyes open and closed conditions. Balance control measures included centre of pressure (COP) root mean square (RMS), COP velocity, and COP mean power frequency (MPF) in anterior-posterior and mediolateral (ML) directions. Trunk sway was estimated using a wearable inertial measurement unit to measure trunk angle, trunk velocity, and trunk velocity frequency amplitude in pitch and roll directions.

**Results:**

The high spasticity group demonstrated greater ML COP velocity, trunk roll velocity, trunk roll velocity frequency amplitude at 3.7 Hz, and trunk roll velocity frequency amplitude at 4.9 Hz, particularly in the eyes closed condition (*spasticity* by* vision* interaction). ML COP MPF was greater in the high spasticity group.

**Conclusion:**

Individuals with high spasticity after stroke demonstrated greater impairment of balance control in the frontal plane, which was exacerbated when vision was removed.

## 1. Introduction

Stroke can result in neurological damage of the upper motor neurons (UMNs) leading to spasticity [1], with studies reporting prevalence of poststroke spasticity of up to 43% [2]. Spasticity is one of several clinical signs of UMN injury defined by Lance as “a motor disorder characterized by a velocity dependent increase in tonic stretch reflexes (‘muscle tone') with exaggerated tendon jerks, resulting from hyperexcitability of the stretch reflex, as one component of upper motor neuron syndrome” [3]. Following stroke, in persons with spasticity, changes in the posture of the upper limbs may result, with excessive internal rotation and adduction of the shoulder, elbow flexion, forearm pronation, wrist flexion, finger flexion, and thumb adduction and flexion [1]. In the lower limbs, spasticity may result in excessive extension, internal rotation and adduction at the hip, plantar flexion and inversion of the ankle, and flexion of the toes [4]. As a result of these changes, it has been well established that individuals with spasticity have great challenges in managing their activities of daily living (ADLs) [1].

A common challenge in persons with stroke is impaired balance control, which affects independence in ADLs [5]. Measures of balance control are related to poststroke falls' incidence [6], and a falls incidence rate of up to 65% has been reported among individuals with stroke [7]. Balance control is complex and involves integration of multiple systems that, when damaged, can result in balance impairment [8]. For example, asymmetry is reported between distribution of forces more on the nonparetic leg in order to maintain standing balance in persons with stroke [9]. In response to external perturbations, persons with stroke demonstrate greater difficulties in maintaining standing balance as compared to healthy and older populations [10], including abnormally low ankle muscle activity on the paretic side [11]. Furthermore, difficulties in voluntarily shifting weight between the feet or relying on other systems that do not involve the affected limbs to maintain balance (i.e., visual system and cognitive control) have also been reported in individuals with stroke [5]. Psychological factors including fear of falling are also important in balance control impairment studies [12], and balance self-efficacy measures have been shown to be an important predictor of balance impairment in persons with stroke [13].

It is established that the action of ankle musculature is important in the control of postural sway during quiet standing, through the action of centre of pressure (COP) movement [14], and that spasticity can result in plantar flexion and inversion of the ankle [4]. Although balance control impairments do exist in individuals after stroke, the role of spasticity in balance control after stroke is unclear [15]. The few recent studies in this area have suggested that quiet standing balance control and interlimb temporal synchronization is impaired among poststroke persons with spasticity compared to those without spasticity [16, 17]. These measures have sufficient sensitivity to detect differences in balance control based on severity of spasticity and to detect changes in balance over the course of recovery [18]. However, the impact of severity level of spasticity on balance control has not been systematically examined.

The focus of the current study was to investigate how severity of spasticity can affect quiet standing balance control in individuals following stroke. It was hypothesized that persons with stroke with high levels of spasticity would demonstrate greater balance control impairment and lower balance confidence measures as compared to poststroke persons with low levels of spasticity. Since balance control is more impaired in absence of vision in persons after stroke [19], it was hypothesized that participants would also demonstrate greater balance control problems during the eyes closed conditions. Hence, it was also hypothesized that individuals with high spasticity would have greater balance impairment in the eyes closed condition in comparison to individuals with low spasticity and conditions with eyes open.

## 2. Methods

Individuals with spasticity after stroke who were receiving treatment in an outpatient spasticity clinic were invited to participate in this cross-sectional study. Inclusion criteria were the ability to stand independently, with and without eyes open for at least 10 minutes. Exclusion criteria were an inability to stand unassisted, inability to follow simple instructions due to cognitive impairments as determined by clinicians, fixed ankle contracture, or being treated with botulinum toxin injections within the past 3 months of study participation. All participants provided informed consent to participate, and this study received ethical approval in accordance with the hospital and university guidelines. Twenty-seven individuals volunteered to participate in the study and met the inclusion/exclusion criteria.

### 2.1. Outcome Measures

#### 2.1.1. Spasticity

The severity of ankle spasticity on the affected limb was determined using the Modified Ashworth Scale (MAS) [18] and scored by a therapist with >7 years of experience in spasticity assessments. MAS has been used as a clinical tool for assessment of spasticity in recent studies of balance control in persons after stroke [16–18]. MAS measures both the nonneural biomechanical and musculotendinous stiffness and the neural component of hyperexcitable stretch reflex [20]. Specifically, MAS is a measure of spasticity (neural component) when no contracture is present; however, MAS measures resistance (nonneural component) to passive movement in the presence of contracture. Although limitations exist in relation to validity and reliability [21], the MAS is a clinically feasible tool [22]. Participants were divided into two groups, high spasticity (*n* = 15) and low spasticity (*n* = 12), based on the severity of ankle spasticity. Sample sizes were slightly different between groups because of the greater prevalence of individuals with high spasticity being treated in the clinic, despite rigorous recruitment methods. In this study, high ankle spasticity was defined as MAS scores of ≥2 in the ankle plantar flexors (i.e., gastrocnemius or soleus) muscles, and low ankle spasticity was defined by MAS scores of <2 in these same muscles. In the spasticity assessments of ankle plantar flexors, subjects were tested in the supine position while testing spasticity in the gastrocnemius muscle was with the knee extended and testing spasticity in the soleus muscle was with the knee in flexed positions. MAS scores in knee flexor, knee extensor, and hip adductor muscle groups of the affected limb were also measured and compared as knee and hip muscle groups are also important in balance control of quiet standing.

#### 2.1.2. Balance Control

A single AMTI force plate (AccuGait Model, Advanced Mechanical Technology, Inc., Watertown, MA, USA) was used to calculate COP as participants stood quietly on the force plate. Trunk sway was simultaneously measured using a wearable inertial measurement unit (SwayStar, Balance International Innovations GmbH, Switzerland), which has embedded angular velocity sensors to measure trunk angular velocity and displacement in the pitch (anterior-posterior) and roll (mediolateral) directions. SwayStar has been shown to provide repeatable, reliable, and sensitive measures in trunk sway of population of individuals with balance problems [23–25].

In each trial, participants stood quietly for 80 seconds on the force plate with the SwayStar system mounted near the lumbar region of the trunk. Participants were instructed not to talk, to remain as still as possible with their feet shoulder width apart, arms hanging by the side, and to look straight at a marker placed on the wall 3 metres ahead during trials with eyes open. For no-vision trials, participants were asked to close their eyes for the duration of the trial. Two clinic staff members stood near each side of participants to stabilize participants in the event of a loss of balance during testing. Each participant completed four trials starting with an eyes open trial for their safety, followed by trials with eyes closed, then eyes open, and then eyes closed. Since the effect of vision is known to affect balance control, the same order of vision testing conditions was used among all participants to keep difficulty level of trials consistent across all participants.

All data, from the force plate and SwayStar, were collected using a sampling rate of 100 Hz. In postprocessing, the first and last 10 seconds of all time-series signals were removed, leaving 60 seconds of data to analyze quiet standing [26]. Residual analysis of raw COP signals was used to determine a filtering cutoff frequency, and COP data were subsequently filtered using a Butterworth low-pass filter cutoff frequency of 10 Hz. COP root mean square (RMS) displacement, COP RMS velocity, and COP mean power frequency (MPF) in the anterior-posterior (AP) and mediolateral (ML) directions were then calculated. COP RMS in AP and ML represents the weighted average of COP displacement in AP and ML, calculated by sum of square of all COP measures in AP and ML divided by total number of samples and taking its square root [27]. COP velocity in AP and ML was achieved by taking the first derivate of COP measures in AP and ML to calculate COP RMS velocity as the weighted average of COP velocity in AP and ML [14]. MPF of COP measures in AP and ML was calculated to analyze COP frequency measures in AP and ML using a Fast Fourier Transform (FFT) algorithm [14].

Angular displacements of the trunk in the pitch and roll directions were calculated online by the SwayStar system software from trapezoid integration of trunk angular velocity measures [28]. Summary measures included 90% percentile range of trunk angular velocity in pitch and roll planes and 90% percentile range of trunk angular deviations in pitch and roll planes. Summary measures also included trunk velocity amplitude calculated based on the square root of spectral densities of trunk angular velocity in the pitch and roll planes and output directly by the system software from 0–20 Hz frequency domains; frequencies were up to 5 Hz (i.e., 1.4 Hz, 2.5 Hz, 3.7 Hz, and 4.9 Hz).

#### 2.1.3. Balance Confidence

The Activities-specific Balance Confidence (ABC) scale was used to estimate balance confidence. Previous research has demonstrated balance confidence to be an important determinant of balance and mobility in population of individuals with spasticity [29] and highly correlated with functional balance measures [30]. The ABC scale has been shown to be a valid and reliable measure in individuals with stroke [31]. The ABC is a 16-item questionnaire that asks participants to rate their confidence based on a percent score from 0% (no confidence) to 100% (completely confident) in each item of the questionnaire. An average score from the 16 questions reflected individuals' balance confidence with lower numbers representing lower confidence [12].

#### 2.1.4. Statistical Analyses

All statistical analyses were conducted using JMP 12 (SAS Institute Inc. 2015.* Discovering JMP 12®*. Cary, NC: SAS Institute Inc.). To test the hypotheses that balance impairments would be greater in (a) the high spasticity group in comparison to the low spasticity group and (b) in eyes closed conditions in comparison to the eyes open conditions, a 2 × 2 mixed model univariate analysis of variance (ANOVA) was performed using the following factors: (a)* spasticity* (high spasticity versus low spasticity) as the between-subject factor and (b)* vision* (eyes open versus eyes closed) as the within-subject factor with JMP's application of Kenward-Roger correction for all COP and trunk sway dependent measures. Tukey's HSD post hoc test was used to further analyze any significant (*spasticity* by* vision*) interaction effects. Least square means comparisons using Tukey's HSD were also used to compare differences in age and ABC scores between high and low spasticity groups.

MAS scores of the hip and knee muscles of the high and low spasticity groups were not normally distributed; therefore, MAS scores between high and low spasticity groups were compared using the nonparametric Wilcoxon rank-sum test. Wilcoxon rank-sum value (*S*) was used instead of a mean value to better represent the MAS scores of muscles with spasticity between groups [32]. Finally, to address the potential order effect inherent in the design of this study, all dependent measure results for trials 1 and 3 (eyes open) and trials 2 and 4 (eyes closed) were compared using Tukey's HSD. A sample size of *n* = 25 was determined as adequate, based on preliminary data of the first six participants, to achieve power of 0.8. For all statistical testing, *α* was set at 0.05.

## 3. Results

### 3.1. Participants' Characteristics

All descriptive statistics' results are reported as least square mean ± SEM (standard error of the mean), except when stated otherwise. Participants' characteristics are presented in Table 1. Distributions of MAS scores in high and low spasticity groups are presented in Tables 2 and 3, respectively.

### 3.2. Balance Control Measures

Statistical analyses of COP and trunk sway measures in AP (pitch) and ML (roll) and their corresponding mean ± SEM values, *F*-statistics, and *p* values are presented in Tables 4 and 5, respectively. The results suggested that individuals with high spasticity had greater balance control challenges during eyes closed conditions (*spasticity* by* vision* interaction) in ML COP velocity, trunk roll velocity, trunk roll velocity frequency amplitude at 3.7 Hz, and trunk roll velocity frequency amplitude at 4.9 Hz measures (Tables 4 and 5). Individuals with high spasticity had greater balance control challenges compared to individuals with low spasticity in ML MPF measures (Table 4). Irrespective of the effect of spasticity, participants had greater balance control challenges in the eyes closed conditions compared to eyes open conditions in AP and ML COP RMS, AP and ML COP velocity, AP MPF, trunk roll angle, trunk pitch and roll velocity, and trunk pitch and roll velocity frequency amplitudes at 1.4 Hz–4.9 Hz (Tables 4 and 5).

### 3.3. Other Findings

There was no significant difference between high and low ankle spasticity groups in ABC scores (*F*(1,25) = 2.35; *p* = 0.14). The MAS scores of the high spasticity group were significantly higher in the gastrocnemius (*S* = 78; *Z* = −4.59; *p* < 0.0001), soleus (*S* = 117; *Z* = −2.73; *p* = 0.0064), knee flexors (*S* = 126; *Z* = −2.15; *p* = 0.032), and knee extensors (*S* = 130; *Z* = −1.92; *p* = 0.05) when compared to MAS scores of the low spasticity group. There was no significant difference between hip adductor MAS scores of the high and low spasticity groups (*S* = 158.5; *Z* = −0.48; *p* = 0.63). Differences in balance control measures observed between spasticity groups in different vision conditions were not influenced by the specific order of testing conditions; dependent measure values were not different between trials 1 and 3 (eyes open) and between trials 2 and 4 (eyes closed).

## 4. Discussion

There is a high incidence of falls in persons after stroke and only a limited number of studies have focused on the role of spasticity in balance control of these individuals [16–18]. The findings of the current study indicate that balance control impairments during standing trials are greater in individuals with high ankle spasticity as compared to individuals with low ankle spasticity and that these differences are exacerbated in the absence of vision. Moreover, these balance control impairments are in the frontal plane, highlighting possible differences in mediolateral balance control of individuals with different level of ankle spasticity after stroke, perhaps premised in the neural and biomechanical changes associated with greater spasticity [20].

### 4.1. COP and Trunk Sway Displacement Measures

There were no differences between high and low ankle spasticity groups in measures of centre of pressure and trunk sway displacement in either the pitch (AP) or roll (ML) directions. These results closely resemble the findings in Singer and colleagues, where it was reported that there were no differences between persons with and without spasticity after stroke in either AP or ML COP RMS [16]. It may be argued that net displacement measures were not different between spasticity groups as individuals with stroke are affected with spasticity only on one side of the body. It is likely that the nonaffected leg with a relatively intact motor control compensated [9] for the high spasticity on the affected leg, resulting in no net change in COP excursion. Indeed, when examining the correlation of centres of pressure between the affected and less affected limbs, a significant reduction in interlimb synchrony is observed in individuals with stroke in comparison to age-matched controls [33] and in poststroke individuals with spasticity in comparison to poststroke individuals without spasticity [16]. These previous findings support the position that net displacement measures may mask balance impairment attributable to asymmetries between the affected and less affected limbs.

### 4.2. COP and Trunk Sway Velocity Measures

In the literature, higher COP velocity values in individuals with stroke [34, 35] and higher trunk sway velocity values in populations of individuals with balance problems [36, 37] generally represent greater balance control difficulties. In the present study, higher mediolateral COP and trunk sway velocity measures observed in the high ankle spasticity group, especially in trials without vision, represent greater mediolateral balance control impairments in this group when compared to individuals with low ankle spasticity. In fact, it has been previously shown that persons with stroke rely more on their vision for balance control during quiet standing as they had greater mediolateral COP velocity measures in absence of vision when compared to healthy elderly individuals [19]. Interestingly, the findings from the current study indicate that individuals after stroke with high ankle spasticity demonstrate similar balance control difficulties when compared to individuals with low ankle spasticity during eyes closed condition.

Current findings may support Horak's explanation of intact somatosensory and visual sensory strategies in balance control of the body [8] and suggest that individuals with high spasticity are more dependent on the surrounding visual cues than individuals with low spasticity in their balance control. Similarly suggested, the proprioception of body position in space helps in balance control [8] and can be altered with higher spasticity levels in persons after stroke [38]. Hence, it can be speculated from balance impairment findings of the high spasticity group in trials without vision that individuals with high spasticity may have poor proprioception that can contribute to greater balance control impairments of this group. The effect of proprioception on balance control of individuals with high spasticity may be tested in future studies as it was not measured in the current study.

### 4.3. COP and Trunk Sway Frequency Measures

In addition to higher trunk sway and COP velocity, the high ankle spasticity group also had higher mediolateral MPF measures than the low ankle spasticity group, regardless of the effect of vision. Higher MPF measures have been associated with greater balance control impairments in populations with balance problems including studies of older versus younger adults [39] and in individuals with stroke versus matched healthy controls [40]. Higher COP frequency measures of the individuals with high ankle spasticity may be explained from an increase in muscle tone. In response to perturbations, rapid reactive responses represented by higher COP frequencies are important for successful balance recovery [17]. Poststroke individuals with balance recovery challenges in response to perturbations have been shown to have greater balance control challenges during quiet standing, specifically in higher frequency ranges (i.e., >0.4 Hz) [41]. Hence, increased frequencies in the high spasticity group in the present study may also suggest inefficient execution of “reactive” balance control strategies in poststroke individuals with high spasticity. Perturbation studies of balance control may further investigate whether individuals with high spasticity levels have greater balance control challenges in their balance recovery. Trunk sway frequency measures similarly suggested higher mediolateral frequency measures in the high spasticity group, more pronounced in absence of vision.

### 4.4. Mechanisms Underlying Velocity and Frequency Differences between Groups

It has previously been demonstrated by Winter and colleagues that the frequency of oscillations during quiet standing movement is proportional to velocity of oscillations which is also proportional to a stiffness measure of a spring model that represents the tone of muscles in control of balance [42]. Hence, an observation of higher mediolateral COP MPF and COP velocity measures in the high spasticity group may be explained by the higher ankle muscle tone, resulting in greater ankle muscle stiffness. Alternatively, a stiffening strategy in response to balance threats, especially in the eyes closed condition, may have been employed by the participants in the high ankle spasticity group [43].

Spasticity is a stretch dependent phenomenon and can be clinically elicited by stretching the affected muscles and the hyperexcitable stretch reflexes are responsible for excessive muscle tone. The natural postural sway, which occurs in both AP and ML directions, can trigger the ankle stretch reflex and in persons with high spasticity can produce excessive increases in tone and enhance the postural sway. We did not see any increase in postural sway in AP direction most likely because the stretch reflex on the nonaffected side may have minimized the impact of the more hyperexcitable stretch reflex on the affected side with high ankle spasticity. Ankle plantar flexor spasticity can also induce invertor moments resulting in equinovarus postures seen in persons with high ankle plantar flexor spasticity [4]. The invertor moments may have been responsible in increasing the ML sway seen in the high ankle spasticity group in the current study. There were no differences found between spasticity groups in the sagittal plane outcome measures. This was surprising as the role of ankle plantar flexors is to control quiet standing balance mainly in the sagittal plane [44] and previously difference in interlimb synchronization of individuals with and without spasticity after stroke in the sagittal plane has been reported [16]. A possible explanation may be that net anterior-posterior measures were not different based on severity of ankle spasticity because all of the participants in the current study were hemiparetic and had no plantar flexors spasticity on the unaffected side. Perhaps the unaffected side contributed enough control in the sagittal plane to compensate for the different levels of spasticity in both groups. Another possible explanation could be the limitation of MAS in discriminating low and high spasticity scores due to possible contractures in the soft tissue around the ankle joint as well as relatively smaller range of motion around the ankle.

### 4.5. Direction-Specific Differences

The current findings show clear differences between groups in the frontal plane. The mediolateral balance control impairments in persons after stroke have been well established in the literature [5], and Winter et al. highlight the role of more proximal muscle groups during quiet standing in mediolateral balance control of the body [44]. In fact, Sosnoff and colleagues similarly found greater mediolateral specific impairments in standing balance control of high versus low ankle spasticity groups in individuals with multiple sclerosis [45]. In their study, Sosnoff et al. too suggested that mediolateral differences between their groups may have been from the action of more proximal muscle groups and speculated that their participants may have also had spasticity in other muscle groups.

In the current study, hip adductor and knee flexor/extensor spasticity scores were assessed. Interestingly, the high ankle spasticity group also had higher spasticity scores in the knee flexor and extensor muscles with no difference in hip adductor spasticity between groups. Higher spasticity in gastrocnemius muscle which spans both ankle and knee joints may have also contributed to the higher knee flexor spasticity of the high spasticity group. It may be possible that higher spasticity in muscles around the ankle and knee joints together could result in greater off-axis moments around these joints, altering the net direction of mediolateral forces and moments that start distally and act proximally towards the centre of mass of the body. Hence, it may be useful to also consider lower limb kinematics and spasticity levels on other muscle groups that are important for balance control in future studies.

We expected that the balance confidence scores would be lower in the high spasticity group; however, no significant difference was observed between balance confidence scores of the groups. It may be that these scores were slightly (but not significantly) higher in the high spasticity group as this group was significantly younger (mean difference: 12.5 ± 4.5 years) and balance confidence scores tend to be typically higher in younger populations [46], though, again, there was no significant difference between balance confidence scores of the groups. Lastly, although balance control is generally less challenging for younger populations, the results of this study suggested that the younger group with higher spasticity had greater balance control impairments. This may further highlight the importance of spasticity level as a considerable factor in balance control of individuals after stroke. Future studies need to compare postural sway in age-controlled groups based on spasticity severity.

## 5. Study Limitations

One limitation of the study was use of a single force platform; therefore, current results cannot further explain the kinetics under each foot. Had this study included use of two force plates, it could have been possible to further investigate the spatiotemporal COP characteristics of the foot with spasticity versus the foot without spasticity in both spasticity groups in addition to net COP characteristics. Severity of spasticity was specifically chosen in the ankle muscles for study design, since standing balance control of the body is mainly under the control of ankle plantar flexors and dorsiflexors with the base of support directly under the ankle joint. Study findings suggested, however, that proximal muscles with spasticity should not be overlooked in better understanding of balance impairments in this population. Limitations in ankle range of motion which may have occurred because of soft tissue changes such as contracture were not measured, and these changes may explain the lack of change in sway in the AP direction. Future studies may also include measures of sensory impairments such as proprioception of the paretic limb between spasticity groups as this was not included in this study. Lastly, the findings of this study are limited to static balance control strategies, whereas dynamic balance control strategies are as important to consider in population of individuals with stroke.

## 6. Conclusion

The results of the current study suggest that individuals with high spasticity have greater balance control impairments in quiet standing, compared to individuals with low spasticity, particularly in the absence of vision. Moreover, the greater postural sway observed in individuals with high spasticity was specific to the mediolateral direction. An increase in muscle tone of the ankle plantar flexors and proximal muscle groups or a stiffening strategy in more difficult balance control tasks can be suggested from findings in individuals with high spasticity. The findings of this study can further be implemented in clinical research and rehabilitation of individuals with spasticity after stroke for a better understanding and treatment of balance control problems in this population. Future studies may also consider the effect of treatment options such as botulinum toxin injections or specific mediolateral balance control training strategies in rehabilitation of individuals with spasticity and balance problems after stroke.

## Figures and Tables

**Table 1:** Participants' characteristics table.

	Low ankle spasticity (MAS < 2)	High ankle spasticity (MAS ≥ 2)
*n*	12	15
Age (years)	74.3 ± 3.4^*∗*^	61.8 ± 3.0
Sex (F/M)	4/8	4/11
Time since stroke (years)	4.4 ± 3.1	9.6 ± 2.8
Affected side (L/R)	5/7	7/8
ABC	57 ± 5.5	68 ± 4.9
Stroke type		
Ischemic	3	5
Haemorrhagic	2	2
Lacunar infarct	1	1
Not available	6	7
MAS (rank-sum “*S*”)		
Gastrocnemius	78^*∗*^	300
Soleus	117^*∗*^	261
Hip adductors	158.5	219.5
Knee flexors	126^*∗*^	252
Knee extensors	130^*∗*^	248

*Note. *Age (mean ± SEM). ABC: Activities-specific Balance Confidence (mean ± SEM); F: female; M: male; L: left; R: right; MAS: Modified Ashworth Scale. ^*∗*^Representing statistically significant difference (*p* < 0.05).

**Table 2:** Distribution of MAS scores in high spasticity group.

Subject	Gastrocnemius	Soleus	Knee flexors	Knee extensors	Hip adductors
1	3	0	1+	2	1
2	3	1+	1+	1+	1
3	3	1+	1+	1+	1+
4	2	1+	0	1	1+
5	2	1+	1	1	1
6	2	1+	1	0	1
7	3	2	1	1+	1+
8	2	1+	1+	1+	1
9	3	2	1+	1+	1+
10	2	1+	0	2	0
11	3	2	2	2	2
12	3	3	1+	2	1+
13	3	2	1+	1+	1
14	2	2	1+	1	2
15	2	2	1	0	1+

**Table 3:** Distribution of MAS scores in low spasticity group.

Subject	Gastrocnemius	Soleus	Knee flexors	Knee extensors	Hip adductors
1	1+	1+	1	0	1
2	1+	1+	1+	1+	1+
3	1+	1+	0	1+	0
4	1+	1+	0	1	1
5	1+	1+	1+	0	1+
6	1+	0	1	1+	1+
7	1	1	0	1+	1
8	1+	1+	1+	1	1+
9	1+	1	0	0	1
10	1+	0	1	0	1+
11	0	1+	0	1+	1+
12	1+	1+	0	0	1

**Table 4:** Dependent measures, *F*-statistics, and *p* values on centre of pressure measures.

	Spasticity		Vision		Spasticity*∗*vision			
	HS	LS	EC	EO	HS/EC	HS/EO	LS/EC	LS/EO
COP AP (mm)	5.1 ± 0.2	5.1 ± 0.3	5.5 ± 0.2^**∗**^	4.7 ± 0.2^*∗*^	5.5 ± 0.3	4.7 ± 0.3	5.5 ± 0.3	4.7 ± 0.3
	*F*(1,24.94) = 0.0002; *p* = 0.98		**F**(1,75.44) = 14.63; **p** = 0.0003		*F*(1,75.44) = 0.016; *p* = 0.90			

COP ML (mm)	3.3 ± 0.3	3.3 ± 0.4	3.5 ± 0.3^**∗**^	3.1 ± 0.3^**∗**^	3.4 ± 0.4	3.1 ± 0.3	3.5 ± 0.4	3.1 ± 0.4
	*F*(1,23.48) = 0.016; *p* = 0.91		**F**(1,70.45) = 5.82; **p** = 0.019		*F*(1,70.45) = 0.097; *p* = 0.76			

COP VEL AP (mm/s)	21.1 ± 2.3	17.1 ± 2.8	22.4 ± 1.9^**∗**^	15.8 ± 1.9^**∗**^	24.9 ± 2.4	17.4 ± 2.4	19.9 ± 2.9	14.2 ± 2.9
	*F*(1,23) = 1.23; *p* = 0.28		**F**(1,73) = 38.88; **p** < 0.0001		*F*(1,73) = 0.72; *p* = 0.39			

COP VEL ML (mm/s)	11.6 ± 1.6	6.9 ± 1.9	10.7 ± 1.2^**∗**^	7.8 ± 1.2^**∗**^	13.7 ± 1.6^*∗*^	9.5 ± 1.6^**∗**^	7.7 ± 2.0	6.1 ± 2.0
	*F*(1,22.83) = 3.63; *p* = 0.069		**F**(1,71.07) = 23.25; **p** < 0.0001		**F**(1,71.07) = 4.73; **p** = 0.033			

MPF AP (Hz)	0.43 ± 0.051	0.36 ± 0.062	0.43 ± 0.041^**∗**^	0.37 ± 0.041^**∗**^	0.46 ± 0.052	0.41 ± 0.052	0.39 ± 0.064	0.33 ± 0.064
	*F*(1,23) = 0.79; *p* = 0.38		**F**(1,73) = 8.68; **p** = 0.0043		*F*(1,73) = 0.069; *p* = 0.79			

MPF ML (Hz)	0.35 ± 0.025^**∗**^	0.27 ± 0.032^**∗**^	0.31 ± 0.023	0.31 ± 0.023	0.36 ± 0.030	0.35 ± 0.029	0.26 ± 0.036	0.27 ± 0.036
	**F**(1,22.61) = 4.52; **p** = 0.045		*F*(1,70.14) = 0.0055; *p* = 0.94		*F*(1,70.14) = 0.19; *p* = 0.67			

*Note.* Values are reported as mean ± SEM; *F*-statistics and *p* values are reported below outcome measures. COP: centre of pressure; VEL: velocity; MPF: mean power frequency; AP: anterior-posterior; ML: mediolateral; HS: high spasticity; LS: low spasticity; EC: eyes closed; EO: eyes open. Bold values connected with the same symbol (*∗*) are significantly different (*p* < 0.05).

**Table 5:** Dependent measures, *F*-statistics, and *p* values on trunk sway measures.

	Spasticity		Vision		Spasticity*∗*vision			
	HS	LS	EC	EO	HS/EC	HS/EO	LS/EC	LS/EO
Pi angle (°)	1.49 ± 0.12	1.58 ± 0.14	1.59 ± 0.10	1.49 ± 0.10	1.57 ± 0.13	1.42 ± 0.13	1.61 ± 0.15	1.55 ± 0.15
	*F*(1,25) = 0.22; *p* = 0.64		*F*(1,79) = 1.75; *p* = 0.19		*F*(1,79) = 0.45; *p* = 0.50			

Ro angle (°)	0.67 ± 0.084	0.66 ± 0.094	0.78 ± 0.067^*∗*^	0.55 ± 0.069^*∗*^	0.80 ± 0.090	0.53 ± 0.094	0.75 ± 0.10	0.57 ± 0.10
	*F*(1,23.6) = 0.002; *p* = 0.96		**F**(1,72.23) = 17.83; **p** < 0.0001		*F*(1,72.23) = 0.67; *p* = 0.41			

Pi VEL (°/s)	1.83 ± 0.15	2.15 ± 0.17	2.23 ± 0.12^*∗*^	1.76 ± 0.11^*∗*^	2.11 ± 0.15	1.55 ± 0.15	2.35 ± 0.18	1.96 ± 0.18
	*F*(1,24.07) = 2.06; *p* = 0.16		**F**(1,74.61) = 50.4; **p** < 0.0001		*F*(1,74.61) = 1.76; *p* = 0.19			

Ro VEL (°/s)	0.82 ± 0.082	0.65 ± 0.10	0.81 ± 0.069^*∗*^	0.67 ± 0.068^*∗*^	0.94 ± 0.086^*∗*^	0.71 ± 0.086^*∗*^	0.68 ± 0.11	0.63 ± 0.11
	*F*(1,23.08) = 1.67; *p* = 0.21		**F**(1,71.18) = 10.84; **p** = 0.0015		**F**(1,71.18) = 4.52; **p** = 0.037			

Pi Amp 1.4 Hz (°/s)	1.69 ± 0.22	2.2 ± 0.25	2.17 ± 0.17^*∗*^	1.72 ± 0.16^*∗*^	1.89 ± 0.23	1.49 ± 0.22	2.46 ± 0.25	1.94 ± 0.25
	*F*(1,23.93) = 2.37; *p* = 0.14		**F**(1,73.35) = 37.65; **p** < 0.0001		*F*(1,73.35) = 0.51; *p* = 0.47			

Ro Amp 1.4 Hz (°/s)	0.85 ± 0.079	0.61 ± 0.097	0.79 ± 0.066^*∗*^	0.67 ± 0.065^*∗*^	0.93 ± 0.083	0.78 ± 0.083	0.65 ± 0.10	0.57 ± 0.10
	*F*(1,23) = 3.71; *p* = 0.067		**F**(1,73) = 10.51; **p** = 0.0018		*F*(1,73) = 0.56; *p* = 0.46			

Pi Amp 2.5 Hz (°/s)	1.02 ± 0.14	1.29 ± 0.15	1.30 ± 0.11^*∗*^	1.02 ± 0.11^*∗*^	1.16 ± 0.14	0.87 ± 0.14	1.43 ± 0.16	1.16 ± 0.16
	*F*(1,24.37) = 1.79; *p* = 0.19		**F**(1,73.74) = 31.91; **p** < 0.0001		*F*(1,73.74) = 0.025; *p* = 0.87			

Ro Amp 2.5 Hz (°/s)	0.41 ± 0.060	0.49 ± 0.067	0.48 ± 0.046^*∗*^	0.42 ± 0.046^*∗*^	0.43 ± 0.063	0.39 ± 0.061	0.53 ± 0.068	0.46 ± 0.068
	*F*(1,24.73) = 0.85; *p* = 0.37		**F**(1,69.57) = 7.99; **p** = 0.0061		*F*(1,69.57) = 0.55; *p* = 0.46			

Pi Amp 3.7 Hz (°/s)	0.61 ± 0.052	0.56 ± 0.064	0.65 ± 0.044^*∗*^	0.52 ± 0.043^*∗*^	0.68 ± 0.055	0.55 ± 0.054	0.62 ± 0.067	0.50 ± 0.066
	*F*(1,21.2) = 0.44; *p* = 0.51		**F**(1,64.38) = 21.47; **p** < 0.0001		*F*(1,64.38) = 0.039; *p* = 0.84			

Ro Amp 3.7 Hz (°/s)	0.31 ± 0.030^*∗*^	0.19 ± 0.038^*∗*^	0.28 ± 0.025^*∗*^	0.23 ± 0.025^*∗*^	0.35 ± 0.032^*∗ ***♯**^	0.27 ± 0.031^*∗*^	0.20 ± 0.040^**♯**^	0.18 ± 0.040
	**F**(1,20.37) = 6.39; **p** = 0.02		**F**(1,62.03) = 13.04; **p** = 0.0006		**F**(1,62.03) = 6.24; **p** = 0.015			

Pi Amp 4.9 Hz (°/s)	0.48 ± 0.069	0.60 ± 0.084	0.61 ± 0.056^*∗*^	0.46 ± 0.056^*∗*^	0.56 ± 0.071	0.40 ± 0.071	0.67 ± 0.087	0.53 ± 0.087
	*F*(1,22.95) = 1.23; *p* = 0.28		**F**(1,72) = 25.68; **p** < 0.0001		*F*(1,72) = 0.063; *p* = 0.80			

Ro Amp 4.9 Hz (°/s)	0.28 ± 0.034	0.20 ± 0.042	0.28 ± 0.028^*∗*^	0.20 ± 0.028^*∗*^	0.33 ± 0.036^*∗*^	0.23 ± 0.036^*∗*^	0.23 ± 0.044	0.18 ± 0.044
	*F*(1,23) = 2.09; *p* = 0.16		**F**(1,73) = 26.07; **p** < 0.0001		**F**(1,73) = 3.51; **p** = 0.065			

*Note.* Values are reported as mean ± SEM; *F*-statistics and *p* values are reported below outcome measures. Pi: pitch; Ro: roll; VEL: velocity; Amp: amplitude; HS: high spasticity; LS: low spasticity; EC: eyes closed; EO: eyes open. Bold values connected with the same symbol ((*∗*) or (*♯*)) are significantly different (*p* < 0.05).
